# Mr. Allen's Case of Phthisis

**Published:** 1803-03-01

**Authors:** R. Allen

**Affiliations:** Colleton, near Kingsbridge, Devon


### *To the Editors of the Medical and Physical Journal.*

Gentlemen,

INDEPENDENT of that which every individual owes to Society, I have no motive for requesting you to give this a place in your useful Journal, than a strong desire of offering my thanks publicly to one, without whose friendly aid the hand which writes this would long since have been in the dust. How far Dr. Beddoes is entitled to this praise of mine, will be best seen by the following relation, which, however inaccurate it may be found in point of composition, is truly correct as to fact. It will likewise prove what merit a certain medical gentleman is entitled to, who has lately taken to himself the credit of introducing the use of calomel in the cure of Phthisis.

For three or four years previous to the autumn of 1800, (the period at which my complaint assumed a threatening appearance) I had been at intervals troubled with a cough, particularly during the winter; however, as it generally disappeared during the summer months, it excited no alarm. At this time, from being troublesome only in the morning, it became likewise so in the evening; and the expectoration, which had hitherto been quite tasteless, became extremely saltish. Though belonging to the medical profession myself, being surgeon of a ship of war, I thought it prudent to take other advice; I accordingly consulted several of the most eminent physicians both in England and Ireland, at different times, and gave to the plans of each more than a fair trial, without deriving the least benefit. And here I must, to the disgrace of medicine, say, that in no one instance was ever the common saying, that "Doctors differ," more completely verified. I was assured by one, that my cough was nervous; by another, that the lungs were in no wise implicated, but that the whole train of symptoms arose from dyspepsia. By one I was advised to try the cold bath, and take antimonials at the same time; another assured me that steel would soon restore me to health; a third, more confident than any of the rest, declared that frequent vomits of ipecacuanha would do the business completely; in this he was perfectly right, for my complaints increased daily.

It may here fairly be asked, how a man in his right senses, putting professional knowledge out of the question,

 could could have adopted such wild and contradictory systems? To this I could have given a much better answer when ill, because my feelings were different. I was content to give up all pretensions to common sense, in hopes of receiving health in return. I had now a fixed pain in the superior part of the sternum, with a constant tickling sensation in the chest; I had no fever, and my appetite remained unimpaired, notwithstanding my flesh wasted by almost imperceptible degrees. In this way I dragged on a miserable existence till the beginning of February, when I was attacked in a most violent manner; the cough became constant, the pain of my chest increased, with pain of the left side; violent fever commenced daily at noon, increasing till towards the morning, when it gave way to profuse colliquative sweats; my pulse, which had hitherto seldom beat more than 60 strokes in a minute, now became full, and increased to 80; the urine also became high coloured and turbid. I again had recourse to the advice of several medical people, not one of whom would allow that mine was a consumptive case; although I had from the beginning conceived it to be an incipient tubercular affection of the lungs, and consequently wished to try the fox-glove, but in this my opinion was completely over-ruled.

In the beginning of April, in a state truly deplorable, all my symptoms increased, and worn to a skeleton, it was my good fortune to turn my thoughts towards Dr. Beddoes; being at Plymouth, and unable to undertake a journey to Bristol, I acquainted him by letter of my situation, and received from him the following answer.

"Dear Sir,

"I have received your very correct statement, and cannot see on what principle you have been refused the use of digitalis. To enter into the pro's and con's would far exceed the limits of a letter; I hope however you will give me sufficient confidence, to make a cautious trial of it. Suppose you begin with five drops of the tincture thrice a day, increasing the dose, so as to guard against its depressing effects; if in the course of ten days you derive no benefit from this plan, I would advise the following: ℞. Tinct. colomb. ʒiij. cin. comp. ʒij. opii ʒiss. M. To a tea spoon full of this compound tincture, which is to be taken thrice a day, add a single drop of digitalis, and three of squill, increasing the dose of digitalis, so as not to exceed half the quantity you found unavailing. I shall be glad to to hear from you in the course of a fortnight, and believe me to be, with best wishes for your recovery,

Your's,

T. B."

It will be unnecessary to say that I scrupulously complied with his directions; at the end of ten days, having taken the digit. to the amount of 25 drops thrice a day, I found no other alteration than that the urine lost its sediment. I then commenced the second plan, and found at the end of five days, that the reduced doses of from 1 to 12 drops of the digitalis, had reduced the pulse to 45 strokes in a minute; my night sweats were considerably lessened; the pain of the chest and side greatly abated; I had still the fever as violent as ever, and my cough was unaltered. Dr. B's. second letter, in answer to mine, stating my partial amendment, is as follows:

"Dear Sir,

"Your letter has given me a mixture of pain and pleasure; but I have little doubt, by calling in all our present resources, you will perfectly recover. Now what I would fain have you try, is a grain and a half, or two grains, of calomel a day, in addition to the digitalis and compound tincture; also a caustic, bigger than a shilling, nearest the place where you suspect most or any disorganization; do not, I conjure you, neglect this. If the skin be hot and dry in the night, rise out of bed, leaving the feet under the clothes; I would almost insure you health on those terms. I am sorry to recommend a complicated plan, but the thing is to restore health, and a little superfluity of medication cannot always be avoided. Write to me when the caustic is in train, for then I shall certainly hear of the reduction of both fever and cough."

The event fully justified his assertions; for in ten days after the application of the caustic, and use of the calomel, the fever was entirely gone, and scarce any cough remained. My amendment henceforwards was so rapid, that, by the beginning of June, I enjoyed as good health as at any period of my life. Conceiving myself perfectly recovered, I incautiously suffered the sore from the caustic to heal, the bad effects of which I soon felt; for, in the latter end of July, being then in the Orion, off Rochefort, I had a considerable return of the cough, with pain of my

 chest, chest. I lost no time in applying a caustic a second time, and adopting the other parts of the plan which had before proved so beneficial, and had the satisfaction to find it soon remove every complaint. I was now determined to act more cautiously, and, by the advice of Dr. B. inserted an issue in the wound made by the caustic, and took, during the winter and spring following, every night, a pill composed of digitalis in powder, opium, and ipecacuanha; the medicine I then omitted, resolving to keep open the issue, which I did until last June, when it became so irritable as to induce me to remove it. Eight months have since gone by without any return of my former illness, less subject than ever to catch cold, without paying the least attention to the circumstances of dress or the weather. The only unpleasant feel I ever experience is during wet and tempestuous weather, when an extraordinary secretion of mucus takes place from the lungs, but which immediately disappears on the return of dry weather.

I should have long since laid this case before the public, had I not preferred waiting the test of time, rather than submitting what might perhaps be considered a doubtful cure. I had another reason besides; whilst I had any thing to expect from the skill of Dr. Beddoes, any thing which I might have said of him might wear the appearance of flattery.

Having nothing now, I trust, to hope from him, what I have now said can only be considered as the effusions of a heart grateful for the recovery of the greatest of all blessings. I cannot, with justice to my feelings, conclude this letter without adding a tribute to his disinterestedness and humanity, as well as his skill; totally unknown to him, and without the prospect of receiving the smallest remuneration for his trouble, his attention exceeded even the anxious wishes of a sick man; those two which I have inserted are not the only letters he has written me on the subject of my health; many, very many, has he been at the trouble of writing, which to publish would only serve more strongly to prove the goodness of his heart, and to spin out a letter I fear much too long already.

In the hope that the above may be found sufficiently interesting to gain an early admission into your Work,

I am, &c.

*Colleton, near Kingsbridge, Devon.*
*Jan.* 18, 1803.

R. ALLEN.

